# A Note on “A Catalog of Biases in Questionnaires” [Response to Letter]

**Published:** 2004-03-15

**Authors:** Bernard C.K. Choi, Anita W.P. Pak

**Affiliations:** Centre for Chronic Disease Prevention and Control, Public Health Agency of Canada, Ottawa, Ontario, Canada; Office of Institutional Research, University of Ottawa, Ottawa, Ontario, Canada

## In Reply:

We would like to thank Dr Harrow (1) for pointing out a flawed example in our paper (2). As he notes, the recommended question should be:

*How many cigarettes do you smoke per day?*[    ] None    [    ] 1–4    [    ] 5–24    [    ] 25 or more

The previously recommended category "4 or less" was a typographical error.

Read the original letter
